# Supplementary material for: From sequence to enzyme mechanism using multi-label machine learning
Source: BMC Bioinformatics. 2014 May 19;15:150. doi: 10.1186/1471-2105-15-150 (PMC4229970; doi:10.1186/1471-2105-15-150)
Supplement: Additional file 2 — Java code of ml2db. Additional file ml2db_code.tar.gz contains the Java source code to run the multi-label machine learning experiments and save the results to database. The code’s Javadoc is included. [file 1471-2105-15-150-S2.zip › additional file 2/ml2db/utils/doc/index-files/index-1.html]

A-Index


JavaScript is disabled on your browser.


- Overview
- Package
- Class
- Use
- Tree
- Deprecated
- Index
- Help

- Prev Letter
- Next Letter

- Frames
- No Frames

- All Classes

A B C D E F G H I J K L M N O P Q R S T U V W X Y 


## A

AbstractContinousDistribution - Class in cern.jet.random
:   Abstract base class for all continous distributions.

AbstractDistribution - Class in cern.jet.random
:   Abstract base class for all random distributions.

acceptEmptyStrings() - Method in class uk.ac.standrews.utils.main.maputils.MapOfMaps


addAll(MapOfMaps) - Method in class uk.ac.standrews.utils.main.maputils.MapOfMaps


addAll(TreeMap<Integer, String>, TreeMap<Integer, String>) - Static method in class uk.ac.standrews.utils.main.maputils.MapUtils
:   Add all key-value entries of map2 into map1 and return map1

addAll(Map<T, U>) - Method in class uk.ac.standrews.utils.main.maputils.OneToManyMap


addAll(OneToManyMap<T, U>) - Method in class uk.ac.standrews.utils.main.maputils.OneToManyMap
:   Add all elements of a one2many map to another map

addChildNode(XmlNode) - Method in class uk.ac.standrews.utils.main.webutils.simpledomparser.XmlNode
:   Adds a child element to the node, and returns the node itself

addKeyAndValue(T, U) - Method in class uk.ac.standrews.utils.main.maputils.OneToManyMap
:   Add a key-value pair to the map

addKeyKeyValue(String, String, String) - Method in class uk.ac.standrews.utils.main.maputils.MapOfMaps
:   Add a key-key-value tuple to the map

addKeyValue(String, String) - Method in class uk.ac.standrews.utils.main.maputils.TableMap
:   Add a key/value set to the one to many map

addStringToArray(String[], String) - Static method in class uk.ac.standrews.utils.main.collectionutils.ListUtils
:   Takes an array and adds a string to all elements

addStringToArrayList(ArrayList, String) - Static method in class uk.ac.standrews.utils.main.collectionutils.ListUtils
:   Takes an arrayList and adds a string to all elements

addSubset(Set) - Method in class uk.ac.standrews.utils.main.collectionutils.setutils.Set


addSubset(Set) - Method in class uk.ac.standrews.utils.main.setutils.Set


AllDatabaseUtilsTests - Class in uk.ac.standrews.utils.test
:   Class

AllDatabaseUtilsTests() - Constructor for class uk.ac.standrews.utils.test.AllDatabaseUtilsTests


AllMapUtilsTests - Class in uk.ac.standrews.utils.test


AllMapUtilsTests() - Constructor for class uk.ac.standrews.utils.test.AllMapUtilsTests


AllStatsUtilsTests - Class in uk.ac.standrews.utils.test.stats
:   Class

AllStatsUtilsTests() - Constructor for class uk.ac.standrews.utils.test.stats.AllStatsUtilsTests


AllUtilsTests - Class in uk.ac.standrews.utils.test
:   Class

AllUtilsTests() - Constructor for class uk.ac.standrews.utils.test.AllUtilsTests


appendPropertiesToFile(File, Properties) - Static method in class uk.ac.standrews.utils.main.FileUtils


appendToFile(File, String) - Static method in class uk.ac.standrews.utils.main.FileUtils
:   Append text to file

appendToFile(String, String) - Static method in class uk.ac.standrews.utils.main.FileUtils
:   Append text to file

appendToFileAndPrint(String, String) - Static method in class uk.ac.standrews.utils.main.FileUtils
:   Append print text to screen (system.out) and append it to file.

apply(double) - Method in interface cern.colt.function.DoubleFunction
:   Applies a function to an argument.

apply(int) - Method in interface cern.colt.function.IntFunction
:   Applies a function to an argument.

apply(double) - Method in class cern.jet.random.AbstractDistribution
:   Equivalent to nextDouble().

apply(int) - Method in class cern.jet.random.AbstractDistribution
:   Equivalent to nextInt().

apply(double) - Method in class cern.jet.random.engine.RandomEngine
:   Equivalent to raw().

apply(int) - Method in class cern.jet.random.engine.RandomEngine
:   Equivalent to nextInt().

arrayFrequencies(Integer[]) - Static method in class uk.ac.standrews.utils.main.stats.StatUtils
:   Records the freqencies with which the integers appear in the array

arrayIsEmpty(String[]) - Static method in class uk.ac.standrews.utils.main.collectionutils.ArrayUtils
:   Checks whether an array is empty

ArrayUtils - Class in uk.ac.standrews.utils.main.collectionutils
:   Utilities to manipulate arrays.

ArrayUtils() - Constructor for class uk.ac.standrews.utils.main.collectionutils.ArrayUtils


AUTO\_INCREMENT\_SQL\_DATATYPE - Static variable in class uk.ac.standrews.utils.main.database.DbUtils


averageArray(double[]) - Static method in class uk.ac.standrews.utils.main.collectionutils.ArrayUtils

A B C D E F G H I J K L M N O P Q R S T U V W X Y

- Overview
- Package
- Class
- Use
- Tree
- Deprecated
- Index
- Help

- Prev Letter
- Next Letter

- Frames
- No Frames

- All Classes
